# Expression of von Hippel–Lindau tumor suppressor protein (pVHL) characteristic of tongue cancer and proliferative lesions in tongue epithelium

**DOI:** 10.1186/s12885-017-3364-8

**Published:** 2017-05-26

**Authors:** Hisashi Hasegawa, Yoshiaki Kusumi, Takeshi Asakawa, Miyoko Maeda, Toshinori Oinuma, Tohru Furusaka, Takeshi Oshima, Mariko Esumi

**Affiliations:** 1 Deparment of Otorhinolaryngology, Head and Neck Surgery, Nihon University School of Medicine, 30-1 Ohyaguchikami-cho, Itabashi-ku, Tokyo, 173-8610 Japan; 2 Department of Pathology, Nihon University School of Medicine, 30-1 Ohyaguchikami-cho, Itabashi-ku, Tokyo, 173-8610 Japan

**Keywords:** Tongue cancer, pVHL, Diagnostic marker, Cytokeratin 13, Cytokeratin 17, Dysplasia

## Abstract

**Background:**

Patients with tongue cancer frequently show loss of heterozygosity (LOH) of the von Hippel–Lindau (*VHL*) tumor suppressor gene. However, expression of VHL protein (pVHL) in tongue cancer has rarely been investigated and remains largely unknown. We performed immunohistochemical staining of pVHL in tongue tissues and dysplasia, and examined the association with LOH and its clinical significance.

**Methods:**

Immunohistochemical staining of pVHL in formalin-fixed, paraffin-embedded sections of cancerous and other tissues from 19 tongue cancer patients showed positivity for LOH of *VHL* in four samples, negativity in four samples, and was non-informative in 11 samples. The staining pattern of pVHL was also compared with those of cytokeratin (CK) 13 and CK17.

**Results:**

In normal tongue tissues, pVHL staining was localized to the cytoplasm of cells in the basal layer and the area of the spinous layer adjacent to the basal layer of stratified squamous epithelium. Positive staining for pVHL was observed in the cytoplasm of cancer cells from all 19 tongue cancer patients. No differences as a result of the presence or absence of LOH were found. Notably, cytoplasm of poorly differentiated invasive cancer cells was less intensely stained than that of well and moderately differentiated invasive cancer cells. pVHL staining was also evident in epithelial dysplasia lesions with pVHL-positive cells expanding from the basal layer to the middle of the spinous layer. However, no CK13 staining was noted in regions of the epithelium, which were positive for pVHL. In contrast, regions with positive staining for CK17 closely coincided with those positive for pVHL.

**Conclusions:**

Positive staining for pVHL was observed in cancerous areas but not in normal tissues. pVHL expression was also detected in lesions of epithelial dysplasia. These findings suggest that pVHL may be a useful marker for proliferative lesions.

**Electronic supplementary material:**

The online version of this article (doi:10.1186/s12885-017-3364-8) contains supplementary material, which is available to authorized users.

## Background

Tongue cancer remains a difficult disease to overcome. Despite the availability of a number of therapeutic modalities and marked advances in techniques to diagnose head and neck cancer, the 5-year survival rate of patients with tongue cancer is approximately 50% [1]. Multimodality therapy combining surgery, radiotherapy, and chemotherapy is generally indicated for advanced tongue cancer. However, in the past few decades, little improvement has been noted in its prognosis.

In general, tongue cancer is more common in older people, but even in the young, the incidence is higher than that of other types of head and neck squamous cell carcinomas (HNSCCs). In addition to chronic stimulation by contact with the teeth and certain environmental factors, alcohol intake and smoking are risk factors for tongue cancer [2], while genetic background also appears to be a strong determinant of risk, particularly in the young [3]. A previous study of genetic abnormalities in HNSCC revealed more frequent loss of heterozygosity (LOH) at loci on chromosomes 3p, 9p, and 17p [4]. Tumor suppressor genes p16 and p53 are located at loci on chromosomes 9p and 17p, respectively, and both are reported to show genetic alterations, such as mutations and methylation, in approximately 50% of tumor specimens from HNSCC patients [5, 6]. Recently, whole exome sequencing of HNSCC revealed that dysregulation of *NOTCH1*, *IRF6*, and *TP63*, which regulate squamous differentiation, is a driver of HNSCC carcinogenesis, similar to mutations of *TP53*, *CDKN2A*, *PTEN*, *PIK3CA*, and *HRAS* [7, 8]. Gross et el. found that a *TP53* mutation is frequently accompanied by loss of chromosome 3p, and that the combination of both events is associated with poor outcomes [9]. Although 3p loss was determined by evaluating 12 genes located in 3p14.2, it remains unclear which factor encoded on 3p is responsible for the interaction with TP53. Asakawa et al. previously demonstrated that LOH of *VHL* (3p25.3), a tumor suppressor gene, occurs at a high frequency in tongue cancer, similar to that of 3p14.2 [10]. However, the biological effect of *VHL* loss on tongue cancer remains unclear.

The *VHL* gene, which is responsible for VHL disease, was identified at loci on chromosome 3p as a tumor suppressor gene in clear cell renal cell carcinoma (RCC) [11–16]. pVHL forms a multimeric complex with Elongin B and C, Culine2, and Rbx1 proteins, which then binds to the α-subunit of hypoxia-inducible factor-1 (HIF-1α) in cytoplasm to induce the ubiquitination and further degradation of HIF-1 [17–22]. HIF-1 induces vascular endothelial growth factor and other angiogenic factors, thereby promoting angiogenesis. Therefore, pVHL serves to negatively regulate angiogenesis. In addition, pVHL is reported to play a role in control of the cell cycle [23].

Here, to clarify the relationship between pVHL expression and the pathology of tongue cancer, we conducted immunohistochemical staining to detect the expression of pVHL in cancer tissues and other lesions from patients with tongue cancer.

## Methods

### Tissue samples

The present study involved 19 patients (eight men and 11 women) with primary tongue cancer, who were treated at Nihon University Itabashi Hospital [10]. Clinicopathological classification of carcinoma and histopathological grading of tumor tissues were based on the Cancer Staging Classification (6^th^ edition) of the International Union Against Cancer [24]. Histological findings of dysplasia included intraepithelial neoplasia lesions lacking infiltration, which were categorized as mild, moderate, and severe dysplasia based on current World Health Organization classifications [25]. Carcinoma *in situ* was not included in the present analysis. For normal tongue epithelium, areas of normal epithelium contained in tissue specimens from patients with invasive tongue cancer were used for investigation. This study was approved by the Ethics Committee of Nihon University School of Medicine (Approval number 118–1). Informed consent was obtained from each patient prior to the start of the study.

### Immunohistochemistry (IHC)

IHC staining was performed using anti-pVHL (556347; BD Biosciences, San Jose, CA, USA), anti-CK13 (NCL-CK13; Leica Biosystems, Nussloch GmbH, Germany), and anti-CK17 (clone E3 IR620; DAKO, Glostrup, Denmark) monoclonal antibodies. Formalin-fixed, paraffin-embedded (FFPE) sections (4-μm thick) of the tissue specimens were deparaffinized in xylene and incubated for 15 min in 5% hydrogen peroxide to inactivate endogenous peroxidases. The treated sections were immersed in 0.01 M citrate buffer (pH 6.0; Muto Pure Chemicals, Tokyo, Japan) and heated for 5 min in an autoclave for antigen retrieval. Each tissue section was then immersed in blocking solution (5% dry skim milk) at 37 °C for 30 min. After removal from the blocking solution, the tissue section was reacted with the primary antibody (anti-pVHL antibody) at a 100-fold dilution in phosphate-buffered saline (PBS) at 37 °C for 60 min. After removal from the primary antibody solution, the tissue section was washed three times (5 min per wash) with PBS.

Chromogenic detection of pVHL was achieved using Histofine Simple Stain MAX-PO (Nichirei Bioscience, Tokyo, Japan) in accordance with the manufacturer’s protocol. For CK13 and CK17, the primary antibody reaction was performed as described above. The subsequent chromogenic detection was performed in two steps: each tissue section was first treated with the Envision Plus kit (EnVision™ FLEX Mini Kit, DAKO) and then with the chromogenic substrate diaminobenzidine (Histofine DAB, Nichirei Bioscience) for 5 min. Each tissue section was counterstained using hematoxylin.

## Results

### Clinicopathological features of tongue cancer

The mean age of the study population was 57.1 (range, 22–79) years. Four subjects were classified as stage I, nine as stage II, three as stage III, and three as stage IV. Fourteen subjects were classified as grade 1, four as grade 2, and one as grade 3. Four specimens were normal epithelium, nine were dysplasia, 16 were well-differentiated carcinoma, six were moderately differentiated carcinoma, and three were poorly differentiated carcinoma. Multiple specimens were obtained from each patient. No somatic mutations of *VHL* were detected in any patient, while four were positive for LOH, four were negative for LOH, and 11 were not informative (Table 1) [10].

**Table 1:** Clinicopathological features of 19 tongue squamous cell carcinomas and immunohistochemistry of pVHL

Case No.	Stage	*VHL* LOH^a^	Grade^b^	pVHL^c^				
				Normal	Dysplasia	Tumor (differentiation)		
						Well	Moderate	Poor
1	II	P	1		A	++	++	
2	II	P	1			++		
3	II	P	1			++		
4	II	P	2			++	++	
5	II	N	1		A	++		
6	IV	N	1			++		
7	IV	N	1	++		++		
8	I	N	1	++	B	++		
9	II	ni	1			++		
10	III	ni	1	++	C	++		+
11	I	ni	1		A	++		
12	I	ni	1	++		++		
13	II	ni	1		A	++	++	
14	I	ni	1			++		
15	II	ni	1		A	++		
16	II	ni	2			++	++	+
17	IV	ni	2		A		++	
18	III	ni	2		A		++	
19	III	ni	3					+
Total				4	9	16	6	3

^a^ LOH, loss of heterozygosity, determined by single nucleotide polymorphism of the *VHL* gene (10). P, positive; N, negative; ni, non-informative

^b^ Grade 1, well-differentiated; grade 2, moderately differentiated; grade 3, poorly differentiated

^c^ All FFPE specimens from 19 cases were examined for histological features and immunohistochemistry of pVHL. All specimens examined were positive for pVHL: ++, strongly positive; +, weakly positive; patterns A, B and C are classifications of dysplasia determined by immunohistochemistry of pVHL together with CK13 and CK17, as shown in Fig. 2b

### IHC staining of pVHL in tongue tissue samples

Upon examination of antigen retrieval conditions using FFPE non-cancerous tissues from clear cell RCC, we detected no staining of pVHL without antigen retrieval (Additional file 1: Figure S1A). Furthermore, trypsin treatment was ineffective for antigen retrieval. Although microwaving was effective, it resulted in strong non-specific positivity. Antigen retrieval by autoclaving facilitated the most intense staining. No pVHL staining was observed using negative control mouse monoclonal antibodies. Proximal tubule staining resembled that obtained with identical antibodies using frozen tissues [26]. Thus, staining for pVHL was performed after heat treatment with pressure. In normal renal tissues, positivity for pVHL was distributed over the entire cytoplasm of cells in the proximal tubule (Additional file 1: Figure S1A). Conversely, in clear cell RCC, the periphery of the cytoplasm was intensely positive with similar findings in frozen tissues of RCC (Additional file 1: Figure S1B).

**Fig. 1:**
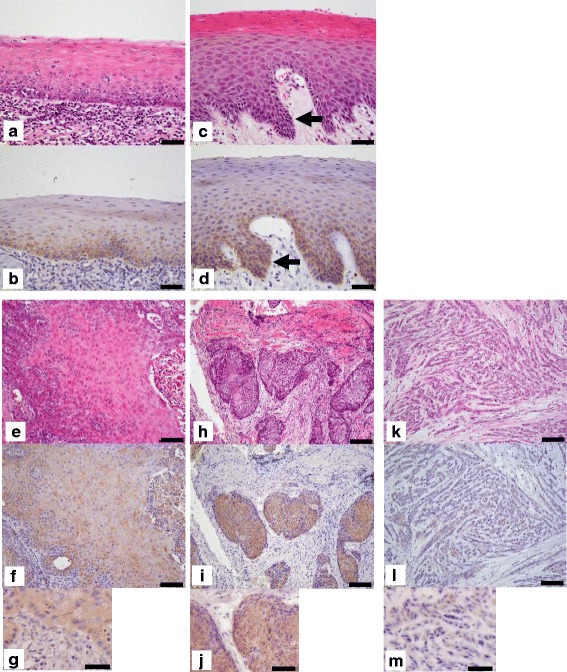
Immunohistochemical staining of pVHL in tongue tissues. Tissues were stained with hematoxylin and eosin (**a**, **c**, **e**, **h**, and **k**), and serial sections were immunohistologically stained for pVHL (**b**, **d**, **f**, **g**, **i**, **j**, **l**, and **m**). **a** and **b**, normal tongue epithelium; **c** and **d**, epithelial dysplasia lesions (*arrows*). Bars indicate 25 μm. **e**, **f**, and **g**, well-differentiated invasive tongue squamous cell carcinoma; **h**, **i**, and **j**, moderately differentiated invasive tongue squamous cell carcinoma; **k**, **l**, and **m**, poorly differentiated invasive tongue squamous cell carcinoma. Bars indicate 50 μm (**e**, **f**, **h**, **i**, **k**, **l**) and 25 μm (**g**, **j**, **m**)

In all specimens of normal tongue epithelium, pVHL staining was localized in the cytoplasm of cells in the basal layer and in parts of the cytoplasm in the spinous layer adjacent to the basal layer (Fig. 1b) (Table 1). No pVHL staining was observed in the stratum corneum or granular layer. In all lesions of tongue dysplasia, pVHL staining was distributed from the basal layer to the middle region of the spinous layer (Fig. 1d). All lesions of invasive tongue cancer were positive for pVHL. The cytoplasm of well-differentiated cancer cells was intensely positive for pVHL in all specimens (Fig. 1f). The peripheral regions of cancerous lesions were more strongly positive for pVHL than the central regions (Fig. 1g). The cytoplasm of moderately differentiated cancer cells was more intensely positive for pVHL than that of well-differentiated cancer cells in all specimens (Fig. 1i, j), whereas the cytoplasm of poorly differentiated cancer cells was more faintly positive for pVHL than that of well-differentiated cancer cells in all specimens (Fig. 1l, m). When the invasion mode and pVHL intensity were compared in cancerous lesions, well-defined cancer tended to be positive for pVHL, and poorly defined cancer was weakly positive for pVHL. However, the relationship was not statistically significant (*p* = 0.059, Pearson’s chi-square test). Staining patterns of pVHL were compared between LOH-positive and -negative cancers for the *VHL* gene. No apparent differences in staining patterns were noted between the positive and negative cases (Additional file 2: Figure. S2).

### Comparison of staining patterns in epithelial dysplasia lesions: pVHL vs. CK13 and CK17

**Fig. 2:**
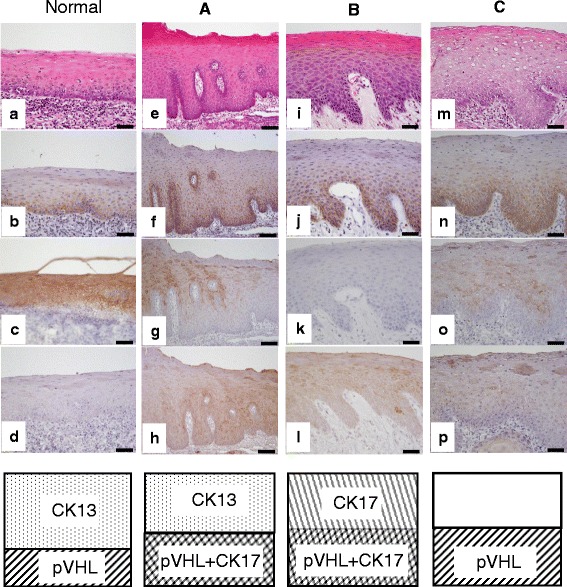
Immunohistochemical staining of pVHL, CK13, and CK17 in tongue epithelial dysplasia lesions. Immunohistochemical staining of epithelial dysplasia. Tissues were stained with hematoxylin and eosin (*first column*), and immunohistologically stained for pVHL (*second column*), CK13 (*third column*), and CK17 (*fourth column*): **a**–**d**, normal tongue epithelium; **e**–**h**, dysplastic epithelial lesions (pattern A); **i**–**l**, dysplastic epithelial lesions (pattern B); **m**–**p**, dysplastic epithelial lesions (pattern C). Bars indicate 25 μm. Schematic patterns of immunohistochemical staining are shown at the bottom. Pattern A, pVHL completely overlapped with CK17; pattern B, normal epithelial cells were positive for CK17 but negative for pVHL; pattern C, dysplastic cells were negative for CK17 but positive for pVHL

The expression patterns of CK13 and CK17 are associated with the development of squamous cell carcinoma and oral epithelial dysplasia. Therefore, these cytokeratins have been suggested to be candidate adjunctive diagnostic markers for oral lesions [27]. In normal tongue epithelium, CK13 staining was observed in the regions that were negatively stained for pVHL (Fig. 2c), but no staining of CK17 was observed in any of the layers (Fig. 2d). Various staining patterns of pVHL, CK13, and CK17 were observed in lesions of tongue dysplasia. We classified the combinations into three categories. Pattern A was characterized by no staining for CK13 and positive staining for CK17 (Fig. 2 g, h) with staining for pVHL largely identical to that for CK17 (Fig. 2f). Pattern A was the typical type and observed in seven of the nine specimens with tongue dysplasia. Pattern B (one of nine specimens) was characterized by no staining of CK13, staining of CK17 distributed throughout all layers (Fig. 2 k, l), and pVHL staining confined to the middle region of the spinous layer (Fig. 2j), which was positive for dysplastic cells. In pattern C (one of nine specimens), CK13 staining was reduced greatly, and CK17 was slightly positive. Although assessment of the atypical grade was difficult for this staining pattern (Fig. 2 o, p), pVHL staining was positive in dysplastic cells (Fig. 2n).

In invasive tongue cancer, we observed no CK13 staining in any of the specimens, and CK17 and pVHL shared positively and negatively stained regions. However, detailed observation revealed that the keratinized regions of well-differentiated invasive cancer were intensely stained for CK17, with no staining for pVHL (Additional file 3: Figure S3).

## Discussion

In this study, we characterized pVHL staining in tongue tissues and cancer as follows. (1) In normal stratified squamous epithelium, pVHL staining was localized to the cytoplasm of cells in the basal layer and parts of the cytoplasm in the spinous layer adjacent to the basal layer. (2) In dysplasia, a precancerous condition, expansion of the range of positivity was observed mainly in dysplastic cells. (3) In invasive cancer, pVHL staining was observed in all specimens, regardless of the differentiation stage. These findings suggest that pVHL will be useful as an adjunctive marker in the histopathological diagnosis of dysplasia.

Here, using our method for pVHL staining, we demonstrated that FFPE sections can be stained with mouse monoclonal antibodies (Ig32) using antigen retrieval procedures. Staining patterns of pVHL in normal renal tissues and clear cell RCC corresponded well with the results of staining using frozen tissue specimens reported by Corless et al. [26]. Claudio et al. [28] stained FFPE sections of clear cell RCC using the same antibody (Ig32) but a different method to ours. A similar staining pattern has been reported using microwaving for antigen retrieval. The present results may be considered as highly reliable. To date, only one other study has reported staining of tongue cancer specimens for pVHL. In that study, 10 of the 27 (37%) tongue cancers were positive for pVHL. Details of this previous study were not well described, but the reason for the substantial difference in the positive rate in our present study remains unclear [29]. However, it is likely a result of the different antibodies used and staining conditions.

To our knowledge, this is the first report of pVHL staining in the basal layer and the spinous layer adjacent to the basal layer of normal squamous epithelium. These results suggest that both stem cells and undifferentiated cells may be positive for pVHL because these cells exist in the same region. pVHL staining has been observed in the cytoplasm of normal epithelial cells in other tissues [26]. In particular, intense staining was noted in renal proximal tubular cells that are considered to be the origin of clear cell RCC [26]. Similarly, tongue cancer appears to develop from abnormal proliferation of stem cells in basal or parabasal cell layers of normal epithelium, which were positive for pVHL.

The clinical condition leukoplakia includes a wide range of lesions, from “reactive” to “precancerous”. Differentiation between reactive and neoplastic tissues is often difficult, particularly in biopsy diagnosis where the observation target is limited to small tissue sections. Our present comparison of IHC staining for CK13/CK17 and pVHL suggests that pVHL staining may be a useful procedure in the evaluation and diagnosis of dysplasia. CK13 and CK17 are useful to evaluate dysplastic grades of certain specimens, such as those with pattern A staining. In pattern B, however, the staining patterns of CK13 and CK17 were typically observed in malignant lesions such as invasive cancer. In contrast, pVHL was stained positively in dysplasia following hematoxylin and eosin (HE) staining. In pattern C, CK13 staining was reduced greatly, while CK17 staining remained slightly positive, a pattern that hampers determination of the dysplastic grade. Nevertheless, pVHL staining was observed in the same dysplastic regions as those stained with HE, and may have superior sensitivity to stain CK13 and CK17 as adjunctive markers to detect dysplastic regions. The present report is the first to investigate the utility of pVHL staining for the diagnosis of tongue dysplasia. However, the small number of specimens examined is a limitation in this study, particularly with regard to dysplasia patterns B and C. An increased number of specimens and another large cohort study are necessary to validate the utility of pVHL in the conclusive diagnosis of preneoplastic lesions and tongue cancer. It would also be helpful to confirm IHC staining of other proliferative markers such as Ki-67 in pVHL-positive dysplasia. Because Ki-67 staining is well correlated with CK17 staining in tongue dysplasia [30], pVHL- and CK17-positive dysplastic regions, at least, may be positive for Ki-67.

Our unexpected finding is that all tongue cancers were positive for pVHL. Because no tongue cancers had nonsynonymous mutations in the present study [10], wild-type pVHL tended to be produced in more differentiated and well-defined cancers. In a HIF-1α-independent pathway, pVHL interacts directly with fibronectin and collagen IV, resulting in their assembly into the extracellular matrix (ECM) and suppression of tumorigenesis, angiogenesis, and cell invasion [31]. Therefore, even in invasive tongue cancer, it is possible that pVHL plays a role in regulation of the ECM and decrease of the invasive ability. Roland et al. demonstrated that poorly differentiated tongue cancers have a poor prognosis [32], and poorly differentiated tongue cancers were weakly stained for pVHL in the present study. In clear cell RCC, pVHL expression is also associated with a low histological grade and better prognosis [33]. Thus, pVHL in cancer possibly functions in the suppression of tumor progression. In the present study, we noted no clear relationship between LOH of the *VHL* gene and the staining pattern of pVHL in tongue cancer. Schraml et al. similarly reported the lack of a relationship between these variables in clear cell RCC [33]. These findings suggest that LOH of the *VHL* gene does not affect expression of pVHL, regardless of the cancer type. Considering the small number of specimens, these results should be considered as preliminary, particularly with regard to dysplasia and poorly differentiated tongue cancers. Therefore, further studies are needed on the topic.

## Conclusions

Regardless of LOH of the *VHL* gene, pVHL was expressed in cancerous and dysplastic tissue in all patients with tongue cancer. These results suggest that pVHL may be a useful adjunctive marker in the histopathological diagnosis of dysplasia.

## Additional files

Additional file 1: Figure S1. Staining of pVHL in clear cell renal cell carcinoma (RCC). (A) Staining of pVHL in normal renal tissues subjected to antigen retrieval. (a) Hematoxylin and eosin (HE) staining, (b–e) pVHL staining, (b) without antigen retrieval, (c) trypsinization, (d) microwaving treatment, (e) heating in an autoclave (arrow indicates proximal tubules), (f) heating in an autoclave (negative control staining with an unrelated monoclonal antibody). Bar indicates 50 μm. (B) Immunohistochemical staining of pVHL in clear cell RCC. (a) HE staining and (b) pVHL staining in clear cell RCC. Bar indicates 50 μm. (PDF 275 kb)

Additional file 2: Figure S2. Comparison of immunohistochemical staining for pVHL between LOH-positive and -negative cases of invasive tongue cancer (well differentiated). (A) Staining of pVHL in an LOH-positive case. (B) Staining of pVHL in an LOH-negative case. Bar indicates 25 μm. (PDF 129 kb)

Additional file 3: Figure S3. Immunohistochemical staining of keratinized regions in well-differentiated squamous cell carcinoma. Tissues stained with hematoxylin and eosin (upper), tissues immunohistologically stained for pVHL (middle), and tissues immunohistologically stained for CK17 (lower). Keratinized regions of squamous cell carcinoma were intensely stained for CK17 (arrows), while the same regions were not stained for pVHL. (PDF 112 kb)

## Abbreviations

CK: Cytokeratin
FFPE: Formalin-fixed paraffin-embedded
HIF-1α: Alpha subunit of hypoxia-inducible factor-1
HNSCC: Head and neck squamous cell carcinoma
IHC: Immunohistochemistry
LOH: Loss of heterozygosity
pVHL: von Hippel–Lindau protein
RCC: Renal cell carcinoma
*VHL*: von Hippel–Lindau gene
