# Supplementary material for: Expression of von Hippel–Lindau tumor suppressor protein (pVHL) characteristic of tongue cancer and proliferative lesions in tongue epithelium
Source: BMC Cancer. 2017 May 26;17:381. doi: 10.1186/s12885-017-3364-8 (PMC5446680; doi:10.1186/s12885-017-3364-8)
Supplement: Supplementary file 1 — Staining of pVHL in clear cell renal cell carcinoma (RCC). (A) Staining of pVHL in normal renal tissues subjected to antigen retrieval. (a) Hematoxylin and eosin (HE) staining, (b–e) pVHL staining, (b) without antigen retrieval, (c) trypsinization, (d) microwaving treatment, (e) heating in an autoclave (arrow indicates proximal tubules), (f) heating in an autoclave (negative control staining with an unrelated monoclonal antibody). Bar indicates 50 μm. (B) Immunohistochemical staining of pVHL in clear cell RCC. (a) HE staining and (b) pVHL staining in clear cell RCC. Bar indicates 50 μm. (PDF 275 kb) [file 12885_2017_3364_MOESM1_ESM.pdf]

A

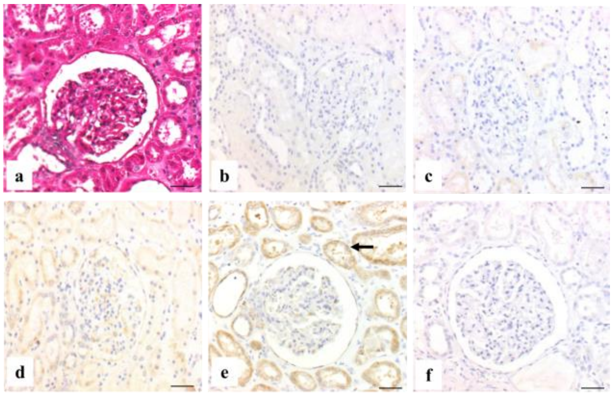


B

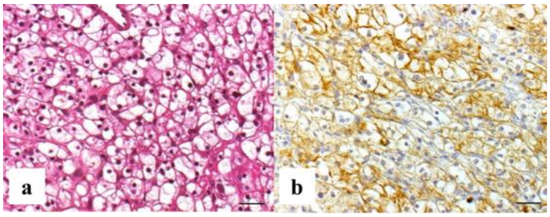


**Figure S1. Staining for pVHL in clear cell renal cell carcinoma (RCC). (A) Staining for pVHL in normal renal tissues treated with antigen retrieval.** (a) Hematoxylin and eosin (HE) staining, (b-e) pVHL staining, (b) without antigen retrieval treatment, (c) trypsinization, (d) microwaving treatment, (e) heating and pressing in a cooking autoclave (arrow indicates proximal tubules), (f) heating and pressing in a cooking autoclave (negative control staining with unrelated monoclonal antibody). Bar, 50 μm. **(B) HE staining for pVHL in clear cell RCC.** (a) HE staining and (b) staining for pVHL in clear cell renal cell carcinoma. Bar, 50 μm.
