# Supplementary material for: Expression of von Hippel–Lindau tumor suppressor protein (pVHL) characteristic of tongue cancer and proliferative lesions in tongue epithelium
Source: BMC Cancer. 2017 May 26;17:381. doi: 10.1186/s12885-017-3364-8 (PMC5446680; doi:10.1186/s12885-017-3364-8)
Supplement: Supplementary file 2 — Comparison of immunohistochemical staining for pVHL between LOH-positive and -negative cases of invasive tongue cancer (well differentiated). (A) Staining of pVHL in an LOH-positive case. (B) Staining of pVHL in an LOH-negative case. Bar indicates 25 μm. (PDF 129 kb) [file 12885_2017_3364_MOESM2_ESM.pdf]

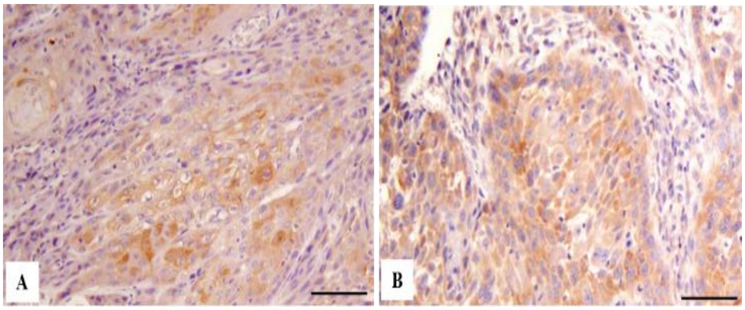

Figure S2. Comparison of immunohistochemical staining for pVHL between LOH-positive and LOH-negative cases of invasive tongue cancer (well-differentiated). (A) Staining for pVHL in an LOH-positive case, (B) staining for pVHL in an LOH-negative case. Bar, 25 μm.
