# Supplementary material for: Expression of von Hippel–Lindau tumor suppressor protein (pVHL) characteristic of tongue cancer and proliferative lesions in tongue epithelium
Source: BMC Cancer. 2017 May 26;17:381. doi: 10.1186/s12885-017-3364-8 (PMC5446680; doi:10.1186/s12885-017-3364-8)
Supplement: Supplementary file 3 — Immunohistochemical staining of keratinized regions in well-differentiated squamous cell carcinoma. Tissues stained with hematoxylin and eosin (upper), tissues immunohistologically stained for pVHL (middle), and tissues immunohistologically stained for CK17 (lower). Keratinized regions of squamous cell carcinoma were intensely stained for CK17 (arrows), while the same regions were not stained for pVHL. (PDF 112 kb) [file 12885_2017_3364_MOESM3_ESM.pdf]

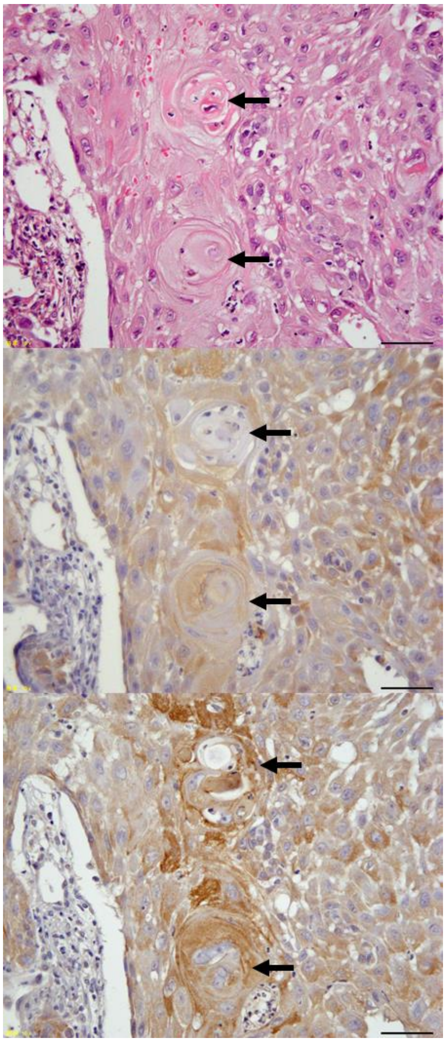

Figure S3. Immunohistochemical staining of keratinized regions of well-differentiated squamous cell carcinoma. Tissues stained with hematoxylin and eosin (upper), tissues immunohistologically stained for pVHL (middle), and tissues immunohistologically stained for CK17 (lower). Keratinized regions of squamous cell carcinoma were intensely stained for CK17 (arrows), while those same regions were not stained for pVHL.
